# Elemental composition of vaping and smoking aerosols: Influence of liquid type and tank conditions

**DOI:** 10.1371/journal.pone.0338087

**Published:** 2025-12-09

**Authors:** Joanna Chwał, Anna Filipowska, Magdalena Antonowicz, Dawid Lisicki, Paweł Kostka, Rafał Doniec

**Affiliations:** 1 Silesian University of Technology, Faculty of Biomedical Engineering, Department of Medical Informatics and Artificial Intelligence, Gliwice, Poland; 2 Joint Doctoral School, Silesian University of Technology, Gliwice, Poland; 3 Academy of Silesia, Department of Clinical Engineering, Katowice, Poland; 4 Silesian University of Technology, Faculty of Biomedical Engineering, Department of Biomaterials and Medical Device Engineering, Gliwice, Poland; 5 Silesian University of Technology, Department of Chemical Organic Technology and Petrochemistry, Gliwice, Poland; 6 The Medical University of Silesia in Katowice, Faculty of Medical Sciences in Zabrze, Department of Materials Science and Artificial Intelligence in Dentistry Science, Katowice, Poland; National Institutes of Health, University of the Philippines Manila/De La Salle University, PHILIPPINES

## Abstract

This study compares the elemental composition of aerosols produced by e-cigarettes and traditional cigarettes using Energy Dispersive Spectroscopy (EDS). Both homemade and store-bought e-liquids were tested under various tank conditions (full, half, and empty), and emissions were collected using nitrocellulose membranes and coil cotton. Additional measurements were performed on unheated e-liquids, cigarette filters, unused cotton, and membranes to assess background contamination. Key elements identified included chromium (Cr), aluminum (Al), sodium (Na), sulfur (S), chlorine (Cl), and copper (Cu). Homemade e-liquids showed significantly higher Cr and Al concentrations, especially under partial tank conditions, suggesting intensified coil degradation. Store-bought e-liquids demonstrated more stable emission profiles. Traditional cigarette smoke contained fewer trace metals but exhibited different elemental patterns. Supplementary viscosity and thermal analyses revealed that homemade liquids degrade more rapidly under heat, which may enhance metal leaching from coils. These findings underscore the influence of liquid composition and device conditions on emission profiles, emphasizing the need for quality control in e-liquid production and further toxicological evaluation of e-cigarette use.

## Introduction

Tobacco use continues to be one of the most pressing public health challenges globally. According to the World Health Organization (WHO), it is responsible for over 8 million deaths each year, with more than 7 million attributed to direct use and around 1.3 million resulting from secondhand smoke exposure [1,2]. Cigarette smoke contains nicotine, tar, carbon monoxide, and hundreds of other harmful substances, many of which are carcinogenic [3,4]. At least 70 of these chemicals are classified as known cancer-causing agents. Cigarette smoking is a major contributor to chronic diseases such as cardiovascular disease, COPD, and lung cancer – which alone accounts for 90% of all cases [2–4]. Despite numerous public health campaigns, legislative action, and nicotine replacement therapies, approximately 1.3 billion people worldwide continue to smoke [1,5]. Within this context, electronic cigarettes, or “e-cigarettes”, have emerged as a widely used alternative to conventional smoking, particularly among younger populations. Marketed as a safer option, these devices work by heating a liquid - typically a blend of nicotine, propylene glycol, glycerin, and various flavorings - into an inhalable aerosol. Proponents suggest that vaping offers a less harmful method of nicotine delivery and may even assist smokers in quitting [6,7]. However, growing evidence highlights the potential health risks associated with e-cigarettes, casting doubt on their reputation as a harm-reduction tool [8,9].

One of the key differences between smoking and vaping lies in the heating mechanism. Traditional cigarettes rely on combustion, with temperatures exceeding 400^°^C, which produces a complex mix of gases and fine particulate matter, including tar and polycyclic aromatic hydrocarbons (PAHs) [10,11]. E-cigarettes operate at lower temperatures (typically between 100^°^C and 300^°^C), generating aerosols through thermal decomposition. While this process avoids many combustion-related toxicants, it can still produce harmful substances such as formaldehyde, acrolein, and metal particles from the heating coil [12–15].

Both products present distinct health risks. Cigarette smoke is well known for containing tar, carbon monoxide, and carcinogens that damage lung tissue, impair oxygen transport, and contribute to chronic inflammation [16–18]. Although e-cigarettes do not emit tar and generate less carbon monoxide, they still deliver nicotine - a highly addictive stimulant that can interfere with brain development, particularly in adolescents and young adults [19]. Additionally, repeated inhalation of vaporized solvents such as glycerin and propylene glycol has been linked to respiratory irritation and impaired lung function. The rise of vaping-related lung injuries, known as EVALI, has further raised alarm, with severe cases resulting in hospitalizations and even fatalities [20,21]. The chemical makeup of traditional and electronic cigarettes may differ, but both contain substances that pose significant health hazards. Cigarette smoke includes high levels of sulfur compounds, cadmium, lead, arsenic, and PAHs [16,22,23]. Sulfur dioxide is a potent respiratory irritant, while cadmium and arsenic are recognized carcinogens that also damage the kidneys and bones [23,24]. Lead, meanwhile, affects nearly every organ system and is especially dangerous to developing nervous systems. Even without combustion, e-cigarettes can expose users to similar toxic elements. The heating coils - often made from metals like lead, nickel, chromium, or aluminum - can release these substances into the aerosol [25–27]. Research shows that users may inhale substantial amounts of these metals, especially under high-heat conditions. In some cases, e-liquids may also contain trace amounts of cadmium, further increasing risk [25,27].

Flavoring agents used in e-liquids pose additional concerns. Compounds such as diacetyl, often added for buttery or sweet flavors, have been linked to bronchiolitis obliterans - a rare but serious disease that scars the small airways and severely restricts breathing [28]. Although e-cigarettes tend to contain fewer hazardous substances overall, the presence of these chemical components indicates they are not without risk.

To better understand what users may be exposed to, researchers have turned to energy-dispersive X-ray spectroscopy (EDS), a technique that allows for the detection of inorganic elements - especially metals - in both cigarette smoke and e-cigarette vapor. Unlike methods that focus solely on organic compounds, EDS provides a broader elemental overview without requiring complex sample preparation. Its sensitivity to a wide range of elements, including trace metals, makes it an increasingly valuable tool in public health research [29–31].

This study provides an integrated assessment of how two major factors – (1) e-liquid composition (homemade vs. commercial) and (2) device operating conditions (full, half, and empty tank) – influence the transfer of trace elements into inhaled aerosol. Using EDS and a simulated lung setup, vapor emissions were evaluated under standardized puffing conditions and compared with traditional cigarette smoke to contextualize exposure risks. Rather than treating elemental emissions, viscosity, and thermal behavior as separate outcomes, these measurements were interpreted together to explain a shared mechanism: increased coil stress and liquid degradation driving metal leaching from chromium- and aluminum-containing heating alloys. In this context, chromium and aluminum were selected as primary analytes due to their direct origin from FeCrAl-based coil materials and their toxicological relevance, while sodium, sulfur, silicon, chlorine, magnesium and calcium were included as additional indicators of liquid composition, coil–wick interaction and thermal degradation processes. Lead and nickel, although commonly discussed in inhalation toxicology, were not chosen as target analytes here because their presence in commercial FeCrAl mesh coils is expected to be minimal; however, both elements remained under observation during analysis to confirm this assumption. While recent studies such as Halstead et al. (2020) [32] have introduced advanced aerosol-trap designs for trace-metal analysis, this study intentionally employs a classical nitrocellulose-based approach to ensure broad accessibility and practical relevance. Limitations related to background contamination and collection efficiency are acknowledged. The working hypothesis assumes that partial depletion of e-liquid enhances thermal stress on the coil and consequently increases the release of metallic species into the aerosol.

## Materials and methods

**Fig 1:**
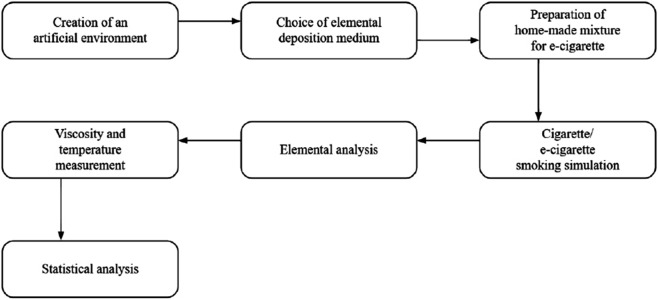
Research stages. The experimental workflow included creation of an artificial environment, choice of elemental deposition medium, preparation of home-made e-liquid, smoking simulation, elemental analysis, and statistical evaluation.

A 0.45 *μ*m nitrocellulose blotting membrane was selected as the collection substrate to simulate lung exposure in a controlled artificial environment. This material, formed by treating cellulose with sulfuric and nitric acid (substituting hydroxyl groups with nitro groups), is highly porous and chemically stable, making it suitable for the deposition and analysis of inhaled elements from both e-cigarette vapor and traditional cigarette smoke. The study focused on heavier elements with known toxicological profiles, including aluminum (Al), copper (Cu), chromium (Cr), sodium (Na), sulfur (S), silicon (Si), calcium (Ca), and magnesium (Mg). These elements are relevant due to their presence in e-liquid ingredients, device components, and potential health risks when inhaled. The e-liquids used included propylene glycol (PG) and vegetable glycerin (VG) in a 50:50 ratio, sourced from Pure Chemical. Pharmaceutical-grade nicotine (Vaping Base) was added to achieve a final concentration of 6mg/mL. Food-grade flavoring concentrates were included to replicate the sensory characteristics of commercial e-liquids. The overall experimental flow is illustrated in Fig 1.

### Creation of an artificial environment

**Fig 2:**
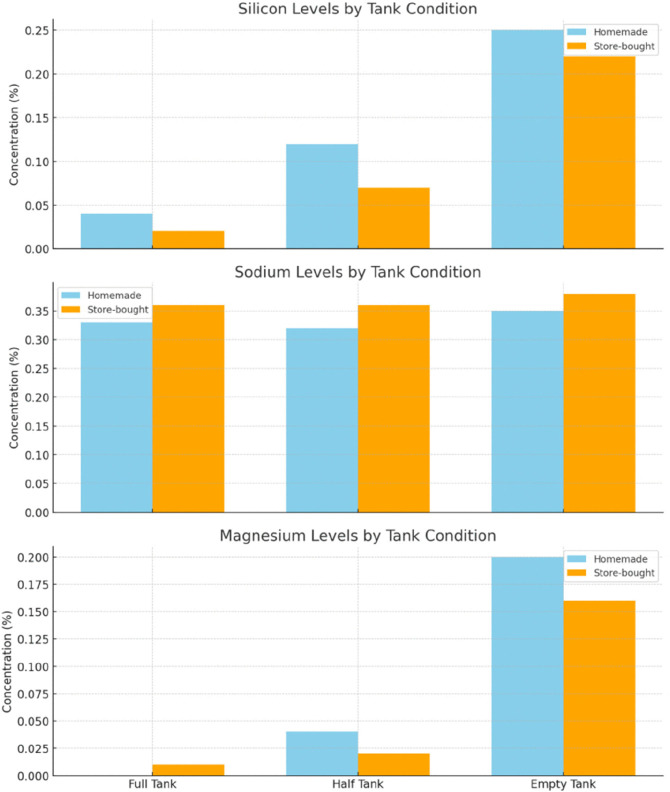
Artificial environment for e-cigarettes testing. The laboratory setup used for controlled generation and collection of e-cigarette aerosol.

**Fig 3:**
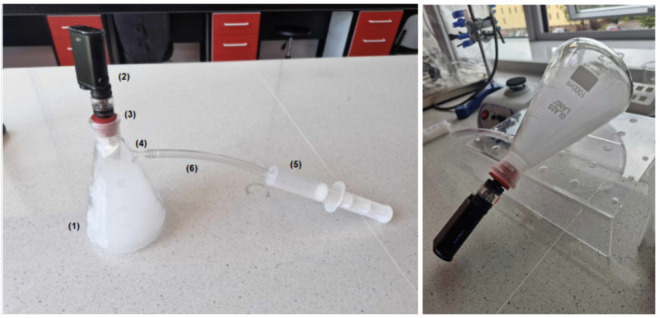
Artificial environment for traditional cigarettes testing. The apparatus designed for standardized generation of conventional cigarette smoke.

A dedicated system was constructed to simulate inhalation and aerosol exposure under laboratory conditions. The setup, shown in Figs 2 and 3, consisted of a conical Erlenmeyer flask (1) sealed with a rubber stopper (3), which housed the e-cigarette device (2). Vapor was introduced into the flask, where it accumulated for analysis. A lateral sidearm (4) was connected to a syringe (5) via flexible tubing (6), allowing manual generation of negative pressure to draw aerosol into the chamber, thereby mimicking human inhalation patterns. Separate configurations were used for testing e-cigarette aerosols and traditional cigarette smoke, ensuring consistent methodology and minimizing cross-contamination.

### Choice of elemental deposition medium

Several deposition media were evaluated to ensure effective collection of aerosol particles. Coal-based filters, initially considered, proved unsuitable due to combustion during testing. Standard filter paper retained large particles (>12–15 *μ*m) but did not capture the full range of emitted elements. Nitrocellulose membranes ultimately provided optimal performance, effectively capturing both particulate matter and associated chemical species. Their physical compatibility with the experimental setup and wide elemental retention range supported their use throughout the study. To further characterize element accumulation, cotton extracted from inside the e-cigarette coil was also analyzed. This material reflects realistic deposition within the device. Following tests involving (i) 10 puffs, (ii) continuous use until coil degradation, and (iii) 100 puffs without refilling, the original coil cotton was carefully removed using sterile tweezers, air-dried, and mounted for elemental analysis.

### Preparation of home-made mixture for e-cigarette

In addition to commercially available e-liquids, a homemade formulation was included to reflect current market behavior, particularly in regions where rising commercial e-liquid prices have led to increasing numbers of users preparing their own liquids. Such products typically contain fewer ingredients and lack proprietary additives such as flavorings or acidifiers, allowing a clearer evaluation of interactions between the base PG/VG matrix and the heating coil. Including both liquid types therefore enables assessment of emissions in realistic consumer scenarios as well as in a more controlled formulation environment.

Homemade e-liquids were prepared using propylene glycol (PG), vegetable glycerin (VG), and pharmaceutical-grade nicotine. A 50:50 PG:VG ratio was used to match common commercial formulations. Nicotine was added to reach a final concentration of 6mg/mL. Initially, 5.78mL of PG and 6.4mL of VG were combined to create the base. Nicotine solution (6mL) was then introduced, followed by 1.82mL of food-grade flavor concentrate to achieve a 10% flavoring ratio. The chosen flavor profile mirrored gooseberry and raspberry notes found in the commercial liquid. All components were measured precisely using volumetric tools. The mixture was stirred thoroughly to ensure full homogeneity, then sealed in an airtight container and stored in a cool, dark location. A 24-hour steeping period allowed the flavors to fully develop and stabilize prior to use. All chemical components used in the preparation of homemade e-liquids, including propylene glycol, vegetable glycerin, and nicotine, were of pharmaceutical-grade (USP), ensuring compliance with established purity and safety standards.

### Cigarette/e-cigarette smoking simulation

All vaping tests were performed using an iStick Pico Plus mod (Eleaf) equipped with a Wotofo nexMINI Subtank atomizer. The heating coil used in the Wotofo nexMINI Subtank atomizer was identified as FeCrAl alloy (Kanthal A1), a widely used material in vaping devices composed of iron (Fe), chromium (Cr), and aluminum (Al). This composition was considered during interpretation of EDS results, especially regarding potential coil degradation products. A 0.2Ω mesh coil was installed, operated at 60 watts – within the manufacturer’s recommended range and typical of regular user settings. This configuration ensured stable aerosol production without overheating or dry hits. Each tank was filled with the test liquid and left to saturate the coil for 10 minutes before activation. The simulation protocol was designed to mirror typical vaping behavior. Each test lasted 5 minutes and consisted of 10 puffs taken at 30-second intervals. Each puff lasted 4 seconds, delivering a consistent volume of 40mL (400mL total). This puffing regimen approximates the consumption of one traditional cigarette, enabling direct comparison of emissions between products. The standardization of puff number, duration, and volume ensured the reproducibility of results and the validity of cross-sample comparisons. For extended-use tests, two additional e-cigarette devices were used to reflect product variability: Voopoo Drag (fitted with a PnP-VM6 0.15Ω mesh coil) and GeekVape Aegis (fitted with a Z-Series 0.2Ω mesh coil). These were employed in normal-use scenarios until coil replacement. The Voopoo coil was composed of nickel-chromium (NiCr) alloy, while the GeekVape coil was made of Kanthal A1 (FeCrAl alloy), similar to the coil used in the Wotofo tank. These models were selected due to their technical similarity, widespread availability in the consumer market, and common use in sub-ohm vaping. Their inclusion allows for realistic simulation of user conditions.

### Elemental analysis

Elemental composition of aerosols and residues was assessed using Energy Dispersive X-ray Spectroscopy (EDS), conducted with an Oxford Instruments Ultim detector. The EDS system was integrated with a TESCAN VEGA 4 scanning electron microscope (SEM), operated under high vacuum using a secondary electron (SE) detector at a working distance of 15mm. Prior to analysis, 5×5cm sections of nitrocellulose membrane were exposed in the collection flask. A central 1×1cm square was then excised and mounted for SEM-EDS to ensure consistency in sampling location. In addition to membrane analysis, post-use cotton from the e-cigarette coils was evaluated to examine trace element accumulation over time. Background measurements were obtained from unused nitrocellulose membranes, unheated homemade and commercial e-liquids, and an unused cigarette filter. These controls were used to distinguish baseline elemental content from exposure-derived contamination. Each measurement was conducted in triplicate (n = 3) per condition. It is important to note that Energy Dispersive Spectroscopy (EDS), while valuable for semi-quantitative screening of elemental composition, has limitations in detecting certain elements at trace levels, especially light or low-concentration metals such as cadmium (Cd) or lead (Pb). Therefore, the absence of these elements in the results does not definitively exclude their presence.

The EDS method was selected because it enables direct identification of trace elements deposited on aerosol-capture substrates and is commonly applied in comparative studies of cigarette and e-cigarette emissions. However, it provides semi-quantitative surface composition rather than airborne mass concentrations or particle size distribution. Therefore, the approach is well-suited for evaluating relative differences between products and usage conditions, but cannot be used to determine absolute inhaled doses. Similarly, the simulated puffing protocol ensures controlled comparability while not fully replicating the variability of real-world user behavior. These aspects are taken into account when interpreting the toxicological implications of the findings.

### Viscosity and temperature measurement

Viscosity is a critical rheological property of e-cigarette liquids, significantly influencing their interaction with the heating coil, vaporization efficiency, and overall performance. Variations in viscosity can impact liquid vaporization rates, aerosol production, and contribute to coil degradation. Therefore, viscosity analysis is vital for comparing formulations and understanding their impact on device functionality.

In this research, dynamic viscosity analysis was performed using a Rheometer type RSCTCPS. Approximately 3g of the sample was placed on the measurement plate, thermostatted with circulating oil at predetermined measurement temperatures ranging from 20 to 80^°^C. The measurements were conducted at four pre-established shear rates (200 to 2000*s*^−1^), providing a comprehensive evaluation of the e-liquid’s rheological behavior.

All samples were tested under identical experimental conditions to ensure accuracy and comparability. The rheological measurements monitored changes in viscosity as a function of temperature, providing insights into how these variations could influence e-liquid performance, particularly regarding vaporization efficiency and interaction with the heating coil.

To supplement the viscosity analysis, temperature tests were conducted to evaluate differences between home-made and commercially available e-liquids. The temperature was measured inside the tank during smoking, without refilling, to reflect realistic usage conditions. A thermocouple type K was used for these measurements, ensuring precise and reliable monitoring of temperature changes within the tank during operation.

### Statistical analysis

Due to the semi-quantitative nature of energy-dispersive X-ray spectroscopy (EDS) data and the limited number of replicates per condition (n = 3; three independent runs denoted I–III), between-group comparisons were performed using the non-parametric Kruskal–Wallis test for independent samples. Two families of comparisons were pre-specified: (i) within-brand comparisons across tank levels (full, half, empty) for homemade and store-bought liquids separately; and (ii) within-level comparisons across liquid types (homemade, store-bought, and tobacco cigarette). The Kruskal–Wallis test was selected as a robust, distribution-free alternative to one-way ANOVA, suitable for small and independent samples of semi-quantitative data. Where appropriate, multiplicity-aware Dunn–Šidák post-hoc contrasts were planned following significant omnibus effects; however, none were ultimately performed, as no Kruskal–Wallis test reached the significance threshold. All analyses were conducted in MATLAB R2025a (MathWorks, Natick, MA, USA).

## Results

The elemental residues produced by both e-cigarettes and traditional cigarettes were analyzed under simulated lung exposure conditions. Aerosol deposits were collected on nitrocellulose membranes, allowing for comparative assessment across product types and usage scenarios. Both homemade and store-bought e-liquids were evaluated under different tank conditions (full, half, and empty), alongside traditional cigarettes. To ensure experimental consistency, the homemade liquid was prepared using commercial-grade ingredients in a standardized 50:50 PG/VG base. Additionally, the study included viscosity profiling of both e-liquid types, as this parameter may influence vaporization dynamics and coil degradation.

Elemental analysis was performed using Energy Dispersive X-ray Spectroscopy (EDS), providing a detailed profile of the chemical constituents present in the aerosols. This approach enabled identification of potential sources of trace elements, including those originating from e-liquid components, heating coil materials, and tobacco combustion. Given the toxicological relevance of many detected metals, such as chromium or aluminum, the findings contribute meaningfully to the assessment of inhalation-related health risks.

To differentiate between background contamination and exposure-induced residues, EDS was also conducted on unused experimental materials. These included: unheated homemade and store-bought e-liquids on a cotton from e-cigarette heating coils, an unused cigarette filter, unused cotton from e-cigarette heating coils, and an unexposed nitrocellulose membrane. The results of this baseline analysis are summarized in Table 1. The clean nitrocellulose membrane exhibited measurable levels of background elements, notably chromium (0.63wt%), sodium (0.59wt%), silicon (0.48wt%), magnesium (0.44wt%), and aluminum (0.33wt%), confirming the membrane’s non-inert character. In contrast, both unheated e-liquids showed only minimal traces (Cu, S, Ca), while the unused filter comprised almost exclusively C and O. Low levels of Cr, Na and Al on coil cotton likely reflect contact with metallic parts during manufacturing. These baselines frame the interpretation of exposure findings that follow.

**Table 1:** Elemental composition (Wt%) and standard deviation (SD) of unheated reference materials. Values are mean ± SD; n = 3. SD reported as 0.00 indicates rounding from very low variance. No inferential statistics were applied to reference materials; these data characterize background composition only.

Element (E)	Nitrocellulose		Home-made liquid on coil cotton		Store-bought liquid on coil cotton		Cigarette filter		Coil cotton	
	wt%	SD	wt%	SD	wt%	SD	wt%	SD	wt%	SD
O	70.4	0.23	58.0	0.10	57.4	0.10	54.7	0.20	50.2	0.10
C	26.4	0.20	41.6	0.10	42.3	0.10	45.2	0.20	48.8	0.10
Cr	0.63	0.07	–	–	–	–	–	–	0.60	0.00
Na	0.59	0.07	–	–	–	–	–	–	0.30	0.00
Si	0.48	0.04	–	–	–	–	–	–	–	–
Mg	0.44	0.06	–	–	–	–	–	–	–	–
S	0.36	0.04	0.10	0.00	0.10	0.00	–	–	–	–
Al	0.33	0.05	–	–	–	–	–	–	0.20	0.00
Cl	0.21	0.04	–	–	–	–	–	–	0.10	0.00
Ca	0.13	0.04	0.10	0.00	0.10	0.00	–	–	–	–

Table notes: “–” indicates element not detected under measurement conditions. Values represent mean weight percentage (wt%) ± standard deviation (SD) for each material type.

Further results from the 10-puff test, corresponding approximately to the consumption of a single traditional cigarette, are presented in Table 2. This test served as a benchmark scenario, allowing for comparative analysis of aerosol emissions from both traditional and electronic cigarettes under standard, controlled usage conditions. Elemental analysis focused on several key elements, including chromium, sodium, aluminum, sulfur, chlorine, silicon, and magnesium. Notable differences in concentration levels between samples offer insight into how these elements behave under combustion, as in tobacco cigarettes, versus vaporization, as in e-cigarettes.

**Table 2:** Average mean elemental concentrations for aerosols produced during single-cigarette or 10-puff tests on a nitrocellulose membrane. Values are mean ± SD; n = 3. SD reported as 0.00 indicates rounding from very low variance. Wt% – weight percentage; SD – standard deviation.

Element (E)	Home-made, full tank		Home-made, half tank		Home-made, empty tank		Store-bought, full tank		Store-bought, half tank		Store-bought, empty tank		Tobacco cigarette	
	wt%	SD	wt%	SD	wt%	SD	wt%	SD	wt%	SD	wt%	SD	wt%	SD
O	54.77	0.62	50.26	0.80	52.00	0.94	53.10	0.71	52.37	0.70	51.94	0.64	48.86	0.35
C	40.40	0.64	40.58	0.88	41.31	1.00	41.12	0.73	41.71	0.75	41.15	0.68	49.89	0.36
Cr	3.84	0.95	6.00	0.97	5.33	0.83	4.81	0.90	4.79	0.86	5.48	0.79	0.33	0.03
Na	0.33	0.03	0.32	0.03	0.35	0.05	0.36	0.04	0.36	0.04	0.38	0.04	0.24	0.03
Al	0.36	0.03	1.40	0.06	0.34	0.04	0.26	0.03	0.21	0.03	0.22	0.03	0.08	0.01
S	0.12	0.02	0.20	0.03	0.10	0.02	0.15	0.02	0.22	0.03	0.22	0.03	0.12	0.02
Cl	0.09	0.02	0.17	0.04	0.12	0.02	0.08	0.02	0.08	0.02	0.14	0.03	0.08	0.02
Si	0.04	0.01	0.12	0.02	0.25	0.03	0.02	0.00	0.07	0.02	0.22	0.02	0.15	0.02
Mg	0.00	0.00	0.04	0.01	0.20	0.04	0.01	0.00	0.02	0.00	0.16	0.02	0.11	0.01
Fe	0.00	0.00	0.72	0.15	0.00	0.00	0.00	0.00	0.00	0.00	0.00	0.00	0.00	0.00
Cu	0.07	0.02	0.06	0.02	0.00	0.00	0.04	0.01	0.04	0.01	0.04	0.00	0.07	0.02
Ca	0.00	0.00	0.00	0.00	0.00	0.00	0.05	0.02	0.02	0.00	0.05	0.01	0.05	0.01
Br	0.00	0.00	0.00	0.00	0.00	0.00	0.00	0.00	0.00	0.00	0.00	0.00	0.04	0.01

Table notes: Elemental composition expressed as mean weight percentage (wt%) ± standard deviation (SD) for each experimental condition (n = 3). SD = 0.00 indicates rounding from negligible variance.

Chromium concentrations were markedly higher in emissions from e-cigarettes, especially in the homemade e-liquid under half-tank conditions (6.00 wt%), compared to 0.33 wt% observed in cigarette smoke. Such enrichment is consistent with intensified coil degradation when the wick is insufficiently saturated, leading to increased release of Cr-containing alloy components into the aerosol. Aluminum levels followed a similar pattern, peaking in homemade liquids with partially depleted tanks (1.40 wt%). Iron appeared only once - in the homemade half-tank group (0.72 wt%) - supporting coil wear as a source rather than e-liquid ingredients. Meanwhile, sodium and sulfur remained relatively consistent across all conditions, suggesting uniform contributions from solvents or additives. Elevated silicon and magnesium in the empty-tank scenario likely reflect thermal breakdown of coil wicking material.

Statistical analysis was performed using the non-parametric Kruskal–Wallis test to compare elemental weight fractions across experimental groups (Table 3). The nominal significance threshold was *α* = 0.05; however, given the exploratory design and limited replication (n = 3), p-values in the range 0.05 ≤ p < 0.10 were interpreted as indicative of emerging trends rather than confirmatory findings. Within the homemade e-liquid group, elemental composition remained broadly stable across tank fill levels (full, half, empty), with no statistically significant differences detected (all p ≥ 0.06). The lowest p-values were observed for magnesium (H = 5.55, p = 0.062) and silicon (H = 4.51, p = 0.105), suggesting a near-threshold trend toward increased emission variability during partial coil depletion. All other analyzed elements – including O, C, S, Cl, Al, Cr, Na, and Cu – showed minimal between-level variation (H ≤ 2.5, p > 0.28).Similarly, in store-bought liquids, the elemental profiles were consistent across fill levels (all p ≥ 0.36), indicating that metal release patterns are largely independent of liquid volume under the tested operating conditions. The absence of significant omnibus effects supports the assumption of experimental reproducibility and ruled out the need for post-hoc contrasts.

**Table 3:** Summary of Kruskal–Wallis test statistics for elemental composition across tank fill levels in homemade and store-bought e-liquids. “–” indicates elements not detected or below the quantifiable threshold. Blockwise trends denote approximate significance in the range of p≈0.05–0.10.

Element (E)	Homemade H statistic	Homemade *p*-value	Store-bought H statistic	Store-bought *p*-value	Blockwise trend (p≈0.05–0.10)
Mg	5.55	0.062	2.03	0.36	–
Si	4.51	0.105	1.57	0.46	4.75 (*p* = 0.093)
Al	0.36	0.84	0.27	0.87	5.54–5.69 (p≈0.06)
Fe	2.00	0.37	–	–	–
O, C, S, Cl, Na, Cr, Cu	≤1.2	≥0.55	≤0.9	≥0.64	–
Ca, Y, Br	–	–	–	–	–

Table notes: Kruskal–Wallis *H* statistics and associated *p*-values summarize nonparametric comparisons across tank fill levels for homemade and store-bought e-liquids. Trends with p≈0.05–0.10 are interpreted as near-significant blockwise patterns rather than definitive group differences.

To complement within-liquid analyses, cross-type comparisons were performed blockwise at each tank level (homemade vs. store-bought vs. tobacco cigarette). None of these Kruskal–Wallis tests reached conventional significance (all p ≥ 0.06), though trends were noted for aluminum in the full- and half-tank groups (H ≈ 5.5–5.7, p ≈ 0.06) and for silicon (H = 4.75, p = 0.093). These subtle patterns may reflect moderate enrichment of aluminum- and silicon-containing residues during coil aging or incomplete evaporation phases.

To assess cumulative effects, elemental analyses were also conducted after 100 puffs and after smoking 10 cigarettes (Table 4). Under these conditions, metal concentrations became more uniform across product types, with chromium stabilizing around 0.20–0.30 wt% and aluminum at approximately 0.10 wt%. This suggests that peak emissions may occur primarily in early-use or insufficient-liquid scenarios, while stabilized conditions reduce the magnitude of coil degradation.

**Table 4:** Average mean elemental concentrations for aerosols produced during 10 cigarettes (100-puff) tests on a nitrocellulose membrane. Values are mean ± SD; n = 3. SD reported as 0.00 indicates rounding from very low variance. Wt% – weight percentage; SD – standard deviation.

Element (E)	Home-made liquid		Store-bought liquid		Tobacco cigarette	
	wt%	SD	wt%	SD	wt%	SD
O	50.30	0.10	49.80	0.10	49.60	0.10
C	48.90	0.10	49.30	0.10	49.60	0.10
Cr	0.20	0.00	0.20	0.00	0.30	0.00
Na	0.30	0.00	0.20	0.00	0.20	0.00
Al	0.10	0.00	0.10	0.00	0.10	0.00
S	0.10	0.00	0.10	0.00	0.10	0.00
Cl	0.00	0.00	0.10	0.00	0.10	0.00
Si	0.10	0.00	0.00	0.00	0.00	0.00
Mg	0.10	0.00	0.00	0.00	0.00	0.00
Fe	0.00	0.00	0.00	0.00	0.00	0.00
Cu	0.00	0.00	0.10	0.00	0.00	0.00
Ca	0.00	0.00	0.00	0.00	0.00	0.00
Br	0.00	0.00	0.00	0.00	0.00	0.00

Table notes: Mean weight percentage (wt%) ± standard deviation (SD) for each condition. SD = 0.00 indicates rounding from negligible variance (very low dispersion).

After extended use (100 puffs or 10 cigarettes), the elemental composition of all aerosols became nearly uniform across product types, indicating stabilization of coil output and minimal ongoing metal release. To further assess the behavior of trace elements over time, additional analyses were conducted on residues collected from cotton extracted from e-cigarette heating coils. These tests followed three usage scenarios: 10 puffs (equivalent to one cigarette), continuous use until coil replacement, and 100 puffs without refilling. The results are summarized in Tables 5,6, and 7.

**Table 5:** Average mean elemental composition after 10 puffs on coil cotton or one cigarette. Values are mean ± SD; n = 3. SD reported as 0.00 indicates rounding from very low variance.

Element (E)	Homemade		Store-bought		Cigarette	
	wt%	SD	wt%	SD	wt%	SD
O	58.61	0.18	59.16	0.19	100.00	0.00
C	41.29	0.18	40.74	0.19	–	–
Cu	0.07	0.01	0.11	0.01	–	–
Zn	–	–	–	–	–	–
Ni	–	–	–	–	–	–
Si	0.05	0.02	–	–	–	–
Cl	–	–	–	–	–	–
Ca	0.07	0.02	0.06	0.01	–	–
Al	–	–	0.05	0.02	–	–
S	0.01	0.00	–	–	–	–
Ti	0.12	0.00	–	–	–	–
Br	–	–	–	–	–	–

Table notes: Elemental composition expressed as mean weight percentage (wt%) ± standard deviation (SD) for each sample type. “–” indicates element not detected or below quantifiable threshold. SD = 0.00 reflects rounding from negligible variance.

**Table 6:** Average mean elemental composition after 100 puffs on coil cotton without refilling. Values are mean ± SD; n = 3. SD reported as 0.00 indicates rounding from very low variance.

Element (E)	Homemade		Store-bought	
	wt%	SD	wt%	SD
O	51.60	0.20	51.30	0.10
C	47.30	0.20	48.30	0.10
Cu	0.20	0.00	0.10	0.00
Zn	–	–	0.10	0.00
Ni	–	–	0.10	0.00
Si	–	–	–	–
Cl	0.00	0.00	–	–
Ca	0.00	0.00	0.00	0.00
Al	–	–	–	–
S	0.00	0.00	0.00	0.00
Ti	0.70	0.00	–	–
Br	0.00	0.10	–	–

Table notes: Elemental composition expressed as mean weight percentage (wt%) ± standard deviation (SD) for aerosols collected after 100 puffs on coil cotton without refilling. “–” indicates element not detected or below quantifiable threshold. SD = 0.00 reflects rounding from negligible variance.

**Table 7:** Average mean elemental composition after normal use, measured on coil cotton (by smoker, until coil change). Values are mean ± SD; n = 3. SD reported as 0.00 indicates rounding from very low variance.

Element (E)	Voopoo Drag (normal-use)		GeekVape Aegis (normal-use)	
	wt%	SD	wt%	SD
O	72.50	0.09	58.77	0.17
C	27.17	0.09	41.17	0.17
Cu	0.16	0.01	0.08	0.01
Zn	0.05	0.01	–	–
Ni	0.03	0.01	–	–
Si	0.03	0.00	0.04	0.01
Cl	0.02	0.00	–	–
Ca	0.01	0.00	–	–
Al	0.01	0.00	0.04	0.01
S	0.01	0.00	–	–
Ti	–	–	–	–
Br	–	–	–	–

Table notes: Elemental composition expressed as mean weight percentage (wt%) ± standard deviation (SD) for residues accumulated on coil cotton after normal use until coil replacement. “–” indicates element not detected or below quantifiable threshold. SD = 0.00 reflects rounding from negligible variance.

When comparing across scenarios, consistent trends emerged. For Cr, homemade liquids exhibited 3.84–6.00 wt% in the 10-puff test (vs. 0.33 wt% in cigarette smoke), but values stabilized near 0.20 wt% (e-cigarettes) and 0.30 wt% (cigarettes) under extended use. Al behaved similarly, peaking at 1.40 wt% (homemade, half-tank) and converging to 0.10 wt% across groups later on. Fe’s single detection (0.72 wt%, homemade half-tank) and absence thereafter points to device-specific wear rather than a sustained emission. Na started slightly higher for store-bought liquids in the 10-puff test (0.36–0.38 wt%) than for homemade (0.32–0.35 wt%) and cigarettes (0.24 wt%), but converged to 0.20–0.30 wt% with extended use – consistent with a reproducible profile driven by base ingredients/additives. S normalized from 0.10–0.22 wt% to 0.10 wt% over prolonged operation, while Cl remained low (0.08–0.17 wt%) without dose-dependent accumulation. Overall, Cr and Al exhibit early peaks under partial/low-tank conditions, whereas Na and S remain comparatively stable. Cotton analyses (Ni, Cu, Zn after prolonged/dry-coil use) reinforce coil stress as the mechanistic driver.

**Fig 4:**
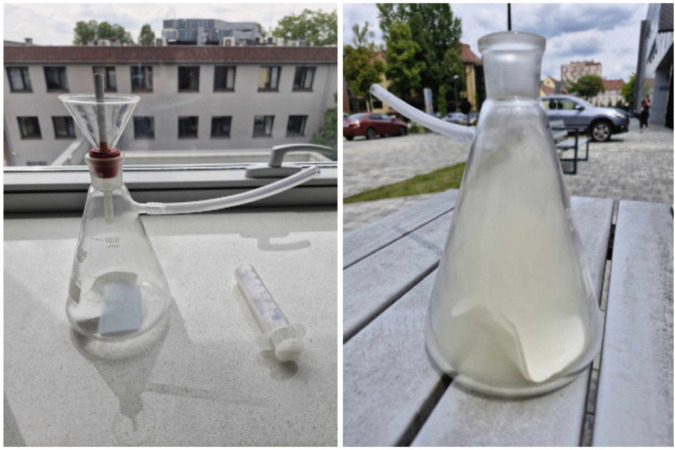
Bar charts of highest concern elements.

**Fig 5:**
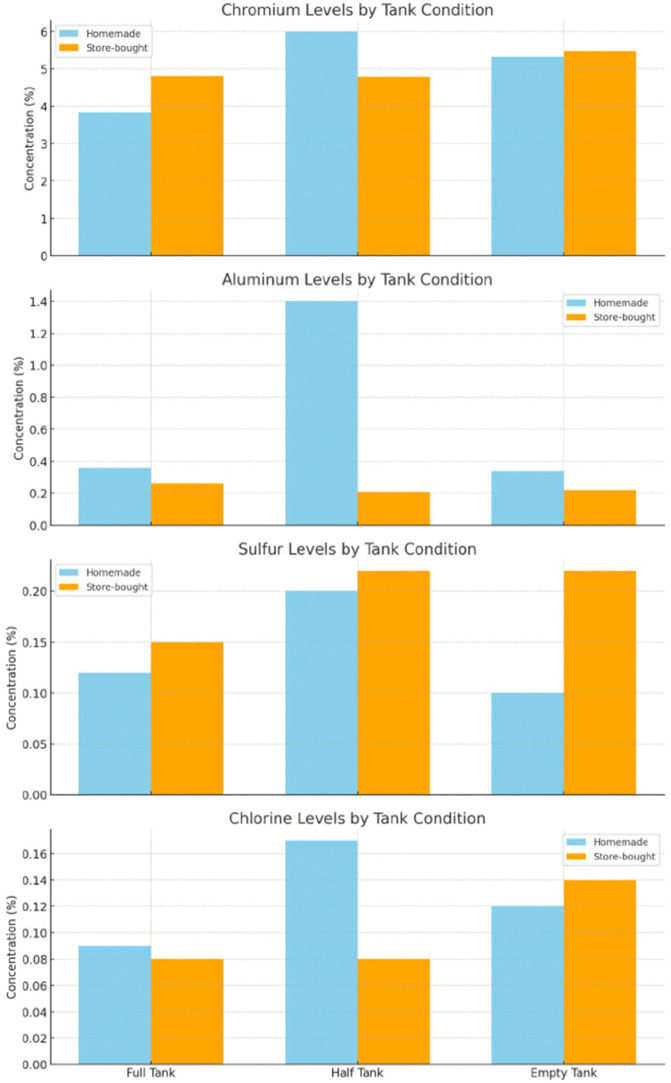
Bar charts of moderate concern elements.

**Fig 6:**
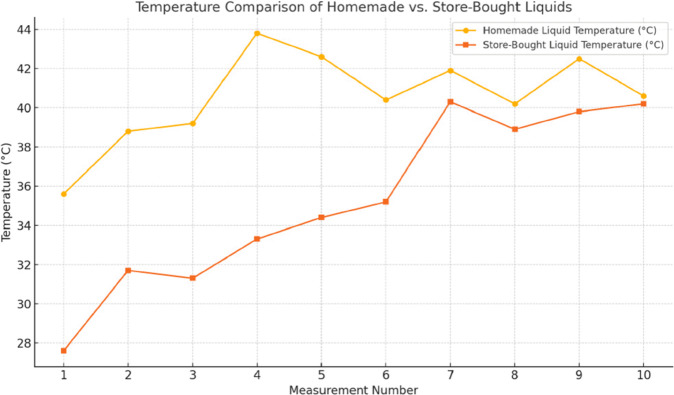
Graphs of elemental composition for homemade and store-bought liquids smoke in comparison to tobacco cigarettes smoke.

To contextualize toxicological relevance, elements were categorized by concern level based on established regulatory criteria, including OSHA permissible exposure limits and WHO air quality guidelines [33,34] (Table 8). Chromium and aluminum were therefore classified as highest-concern metals due to their recognized carcinogenic and neurotoxic potential, respectively, and were monitored closely for condition-dependent changes. Lead and nickel – although widely discussed in inhalation toxicology – are not expected in FeCrAl mesh coils and were therefore not included as primary analytes, but remained under observation throughout the analysis. Their distributions across tank levels/liquid types are visualized in Figs 4,5, and 6. In brief, Cr and Al – highest-concern metals – show the clearest sensitivity to tank depletion, with homemade liquids displaying larger half-tank excursions and both liquid types increasing under empty-tank conditions. Store-bought liquids exhibited more stable profiles overall, though S and Cl were consistently higher in some store-bought conditions, plausibly reflecting additive chemistry. Si and Mg increased under empty-tank operation, consistent with wicking material degradation. These figure-based trends are concordant with the tabulated results above and the coil-stress mechanism.

**Table 8:** Categorization of elements by concern level in aerosol composition analysis.

Highest concern elements	Moderate concern elements
Chromium (Cr)	Silicon (Si)
Aluminum (Al)	Sodium (Na)
Sulfur (S)	Magnesium (Mg)
Chlorine (Cl)	–

Table notes: Classification based on potential toxicological relevance and detection frequency across experimental and normal-use conditions. “Highest concern” elements are those with known or suspected health implications when aerosolized; “moderate concern” elements showed lower concentrations or less consistent occurrence.

The Fig 4 bar charts show key trends in elemental concentrations across different e-liquid types and tank conditions. Chromium levels are slightly more stable in store-bought e-liquids, but both types increase in the empty tank. Homemade e-liquids have significantly higher aluminum levels in the half-tank condition compared to store-bought ones. Store-bought e-liquids consistently show higher sulfur levels, especially in the half and empty tanks. Chlorine is higher in homemade e-liquids in the half-tank condition, while store-bought e-liquids see an increase in chlorine in the empty tank, possibly due to coil degradation or additive residues.

The Fig 5 bar charts reveal trends in silicon, sodium, and magnesium concentrations across different e-liquid types and tank conditions. Homemade e-liquids show higher silicon levels in the empty tank, while store-bought e-liquids are more consistent but increase significantly in the same condition. Store-bought liquids generally have slightly higher sodium levels with minimal variability across tank conditions. Homemade e-liquids exhibit a noticeable magnesium increase in the empty tank, while store-bought liquids show a smaller rise but still increase in this condition.

**Fig 7:**
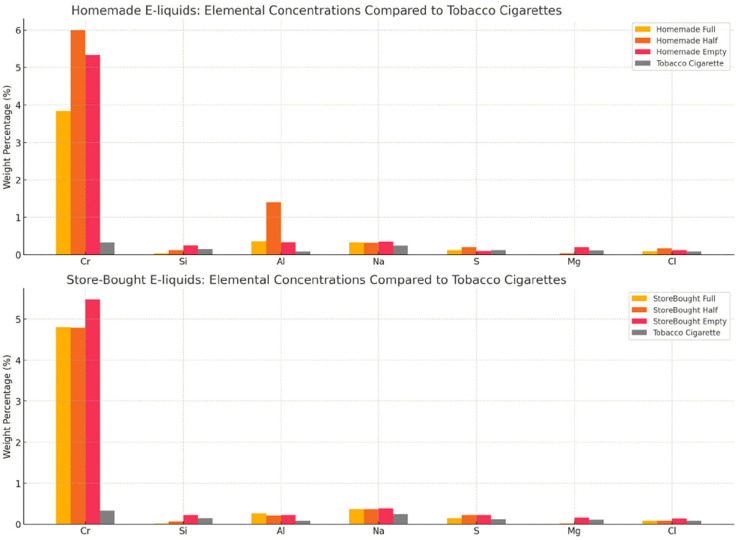
Viscosity results.

**Fig 8:**
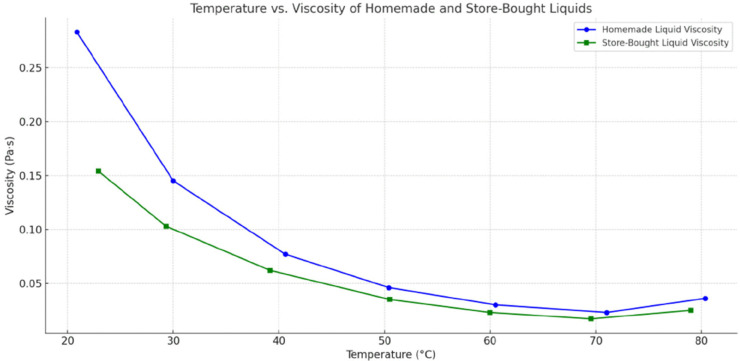
Temperature results.

Because liquid rheology modulates wicking and heat transport, we profiled viscosity as a function of temperature (Fig 7). The homemade liquid started at ∼0.2848 Pa⋅s (≈20.9 ^°^C) and dropped sharply to ≈0.0295 Pa⋅s (≈60.5 ^°^C), with a slight uptick at 80 ^°^C (0.036 Pa⋅s), indicating onset of thermal degradation. The store-bought liquid began lower (∼0.15 Pa⋅s at ≈23.5 ^°^C) and declined more gradually, consistent with stabilizers improving thermal behavior; a small rise at 80 ^°^C (∼0.025 Pa⋅s) likewise suggested incipient degradation. Shear-stress readings tracked these differences (homemade ≈56–57 Pa at ∼200 *s*^−1^ vs. store-bought ≈30–34 Pa). Complementary in-tank thermometry (Fig 8) showed faster initial heating and higher early peaks for the homemade liquid, but both systems stabilized around ∼40 ^°^C under the tested regimen. Taken together, rheology and temperature profiles provide a mechanistic bridge: partial depletion and reduced wicking elevate local coil temperature/stress, facilitating metal leaching (Cr, Al) observed in the short-term, half/empty-tank conditions, with attenuation once operation stabilizes or liquid is replenished.

## Discussion

This study aims to provide a qualitative assessment of the elemental composition of inhalation products from electronic and traditional cigarettes, using a simulated lung exposure model to better understand their potential health effects. Although aerosol mass and dose per lung surface area were not quantified, puff count and volume were standardized to approximate typical usage conditions and allow relative comparison between products. The results show significant differences in the elemental profiles of emissions from e-cigarettes (with homemade and store-bought liquid) compared to traditional cigarette smoke, highlighting the complexity of potential risks associated with e-cigarette use. The observed differences in elemental emissions, such as the presence of aluminum, chromium, sodium, sulfur and other metals, underscore the unique chemical composition of e-liquids and highlight the potential health risks that can result from inhaling these substances. While the concentrations of these elements are relatively low, the cumulative effect of long-term exposure through regular e-cigarette use is not well understood. The nitrocellulose membrane itself was found to contain multiple trace elements, including chromium, sodium, and aluminum, prior to exposure. This highlights the limitations of traditional membrane-based collection methods for trace metal analysis. Consequently, complementary analysis using cotton from within the heating coil provided a cleaner and more relevant sampling medium, allowing for validation of the deposition patterns observed on the nitrocellulose substrates.

Recent research into the particulate matter from e-cigarettes has underscored the need for comprehensive investigations into their impact on human health. Fernández et al. (2015) conducted a comparative analysis of particulate emissions from electronic and traditional cigarettes, revealing that e-cigarette aerosols, while less chemically complex, may still deposit harmful particles in the respiratory tract [35]. Similarly, Pichelstorfer et al. (2020) highlighted the anatomical variability in particle deposition within the lungs, suggesting differential health impacts between devices [36]. Moreover, the thermal behaviour of e-cigarette liquids has been shown to influence aerosol composition. Lampos et al. (2019) found variations in emissions depending on the device and liquid characteristics, with significant impacts on the concentration of harmful constituents like heavy metals [37]. Szparaga et al. (2021) reviewed the chemical content of aerosols generated by e-cigarettes, highlighting the presence of potentially harmful metals and thermal by-products that may exacerbate health risks [38].

The combined analysis of viscosity and temperature highlights critical differences in the operational behavior of homemade and store-bought e-liquids, with direct implications for both user safety and device performance. E-liquids with higher and more stable viscosity tend to ensure consistent vaporization and reliable wicking, reducing localized overheating and subsequent coil degradation. In contrast, liquids of lower or highly variable viscosity may lead to insufficient saturation of the coil, accelerating wear and increasing the likelihood of trace metal release into the vapor. In this study, homemade liquids demonstrated greater viscosity variability across the tested temperature range, which may contribute to inconsistent aerosol formation and transient exposure spikes. Meanwhile, store-bought e-liquids generally maintained more uniform viscosity and heating behavior, minimizing abrupt thermal changes, stabilizing coil operation, and potentially lowering exposure to harmful elements. These observations are consistent with Ko et al. (2022) [39], who demonstrated that both viscosity and coil temperature govern thermal stress within the heating assembly and can influence the emission of metals and other degradation products. It is important to note that relatively stable viscosity of homemade liquids at sustained operating temperatures does not necessarily translate into reduced metal emissions; rather, the early-phase instability during partial tank depletion remains the dominant driver of coil stress and trace-metal release.

The presence of trace elements like chromium, aluminum, and sodium in e-cigarette emissions, as observed in this study, aligns with findings from other recent research. Additional evidence came from direct analysis of coil cotton, where elevated levels of chromium, copper, and nickel were detected after extended use, supporting the hypothesis that these metals originate primarily from coil degradation rather than e-liquid composition. Studies have shown that e-cigarette aerosol contains various metals, likely originating from the coil material and heating process, which may pose inhalation risks. For example, a study by Aherrera et al. (2023) [40] found that e-cigarette aerosols contain significant levels of metals, with some reaching concentrations high enough to be concerning for users’ respiratory health. In this study, elevated levels of chromium and aluminum were observed in certain e-liquid samples, particularly in homemade e-liquids under the half-tank configuration. These metals are known for their toxicity, and prolonged exposure could contribute to respiratory and neurological diseases. The chromium in e-cigarettes, as highlighted by the research of Aherrera (2017) [41], further reinforces concerns about the potential carcinogenic effects of hexavalent chromium, which could be released during vaping.

In this study, while traditional cigarettes also release a variety of toxicants, the elemental concentrations for metals like chromium and aluminum in tobacco smoke were not as pronounced as in e-cigarettes. This aligns with the findings of studies like that of Williams et al. (2013) [42], which reported that cigarette smoke contains various toxic compounds, but metals like chromium were not as prevalent when compared to e-cigarette aerosols. However, during extended operation (100 puffs / 10 cigarettes), the differences between product types diminished, indicating that metal exposure from e-cigarettes is highly condition-dependent and primarily elevated under partial-tank scenarios. Thus, while cigarettes remain far more hazardous overall due to combustion-derived toxicants, e-cigarettes may exceed them specifically in metal-based exposure under certain device conditions. Tobacco combustion produces a mix of thousands of chemicals, many of which are carcinogenic, but the heating mechanism in e-cigarettes may contribute to the increased release of metals and other compounds like sulfur and chlorine, which are less common in traditional cigarette emissions. Notably, lead and cadmium - commonly scrutinized in inhalation toxicology - were not detected in the tested samples. This could result from either their actual absence or limitations in detection sensitivity of the EDS technique. Nickel, another toxicologically relevant metal, was detected in both aerosol residues and coil cotton samples, although not consistently across all devices. Notably, nickel was present in the aerosol sample collected after 100 puffs using the iStick Pico Plus with a FeCrAl (Kanthal A1) coil, despite this alloy not typically containing nickel. This suggests possible trace contamination from manufacturing, solder joints, or contact materials. In contrast, nickel was also observed in post-use coil cotton from the Voopoo Drag device, which uses a NiCr coil – supporting the hypothesis that coil composition contributes to nickel exposure, though not exclusively.

A unique contribution of this study is the evaluation of tank-level conditions (full, half, empty), which revealed that metal concentrations, particularly chromium and aluminum,increased under partially depleted liquid scenarios, especially for homemade e-liquids. This observation is consistent with Gordon et al. (2021) [43], who reported increased emission toxicity during tank depletion due to overheating and coil degradation, and with Aherrera et al. (2023) [40], who linked sulfur- and chlorine-containing additives in commercial e-liquids to enhanced release of harmful by-products upon heating. Collectively, these findings emphasize that trace elements originating from e-cigarette hardware and liquid composition may pose chronic health concerns. Chromium, especially in its hexavalent form, has been associated with respiratory toxicity and cancer [44,45], while prolonged exposure to aluminum has been implicated in neurological disease, including Alzheimer’s [46]. Thus, operational conditions such as coil material and tank fullness – and not only e-liquid ingredients – may influence the magnitude of inhalation exposure and the associated health risks.

Recent regulatory evaluations, including those by the FDA and European agencies, have acknowledged that certain ENDS products may be appropriate for public health protection under specific conditions (Bolt, 2024 [47]). However, concerns regarding exposure to trace metals and the long-term health implications of these devices remain significant. The coil used in this study was a standard 0.2 Ω mesh coil, typically made of FeCrAl alloy (Kanthal A1), which includes iron, chromium, and aluminum. These materials are common in commercially available vaping devices and are known sources of metal particulate contamination during heating. The inclusion of used coil cotton analysis provided a complementary dataset, which helped validate the deposition findings from nitrocellulose membranes by confirming the presence of similar trace elements at the vaporization source. Lead (Pb) and cadmium (Cd) were not detected in the tested samples, which may be due to differences in coil composition, liquid purity, or the detection limits of the analytical method used. Nickel (Ni), while not consistently found across all scenarios, was observed in specific coil cotton samples, supporting its origin from metal components rather than e-liquid content.

To contextualize the measured concentrations, we briefly summarize health concerns and reference limits associated with the key elements detected. Chromium (Cr), particularly Cr(VI), is a known carcinogen; prolonged inhalation elevates respiratory and cancer risk (OSHA PEL 5*μ*g/m^3^, 8-h TWA) [33]. Aluminum (Al) exposure has been linked to neurotoxicity and respiratory effects (OSHA PEL 15mg/m^3^ total dust, 5mg/m^3^ respirable) [33]. Chlorine (Cl) is a potent airway irritant (OSHA PEL 1ppm, 8-h TWA) [33]. Sulfur compounds can exacerbate asthma; the WHO recommends a 24-h mean limit of 40*μ*g/m^3^ for SO_2_ [34]. Silicon (Si) is a concern primarily in crystalline silica form (OSHA PEL 50 *μ*g/m^3^ respirable) [33]. By contrast, sodium (Na) and magnesium (Mg) are generally of lower concern at the trace levels detected here. Importantly, our EDS data are semi-quantitative and do not provide airborne mass concentrations; therefore, these limits are cited for context rather than direct risk quantification. Future work should convert deposition signals to inhalation doses to enable formal risk assessment.

While the present study emphasizes elemental exposure risks, it is also important to consider the broader regulatory context. Recent product-specific authorizations by the U.S. FDA acknowledge that under certain conditions, some ENDS products may be appropriate for the protection of public health. However, concerns regarding coil degradation and long-term trace metal exposure remain unresolved. Additionally, although EVALI has been primarily linked to illicit THC products, recent reviews such as Bolt (2024) [47] underscore the importance of continuous monitoring of all vaping devices.

Several methodological constraints must be acknowledged when interpreting these results. First, the EDS-based approach provides semi-quantitative surface composition rather than inhaled dose or aerosol mass concentrations, which limits direct comparison to established toxicological thresholds. Second, only two commercially available device types and selected coil materials were examined; thus, findings may not fully represent the diversity of ENDS products on the market. Third, nitrocellulose membranes contained inherent trace-element background, although complementary coil-cotton sampling helped validate exposure-driven deposition. Finally, the simulated puffing protocol approximates typical use but does not capture user-specific behaviors such as chain vaping or device power modifications. Future investigations should incorporate broader device and liquid variability and use quantitative dosimetric methods to assess exposure and health-risk relevance under real-world vaping conditions.

## Conclusion

This study demonstrates that e-cigarette aerosols contain measurable levels of trace metals originating primarily from coil materials, and that these emissions can vary substantially depending on liquid formulation and tank fill level. Under conditions of reduced liquid availability, particularly half or nearly empty tanks, metal release increased, indicating that user behavior and device maintenance critically influence exposure. Although traditional cigarettes remain hazardous due to their complex toxicant profile, the present results show that e-cigarettes are not free of inhalation risks and may in some scenarios produce higher levels of selected elements such as chromium and aluminum.

These findings indicate a need for careful regulation of device materials and liquid formulations, as well as user guidance to avoid high-temperature operating conditions associated with coil degradation. Future studies should quantify deposited metal mass to enable dose-based risk assessment, validate the semi-quantitative EDS findings using complementary techniques such as ICP-MS, and extend testing to a broader range of devices and real-world vaping behaviors. Taken together, this work reinforces that e-cigarette safety is highly dependent on usage conditions and product design, and continued evaluation is essential to reduce potential long-term health risks.

## Supporting information

S1 Data Elemental composition. (XLSX)

S2 Data Viscosity. (XLSX)
